# Efectividad de la prevención terciaria en la calidad de vida y control de los factores de riesgo en pacientes con cardiopatía coronaria isquémica

**DOI:** 10.47487/apcyccv.v4i3.323

**Published:** 2023-09-30

**Authors:** Rosalía Fernández Coronado, Adriel Olórtegui Yzu

**Affiliations:** 1 Instituto Nacional Cardiovascular INCOR, Lima, Perú Instituto Nacional Cardiovascular INCOR Lima Perú

**Keywords:** Prevención Terciaria, Rehabilitación Cardiaca, Calidad de Vida, Factores de Riesgo de Enfermedad Cardiaca, Tertiary Prevention, Cardiac Rehabilitation, Quality of Life, Heart Disease Risk Factors

## Abstract

**Objetivo:**

. Determinar la efectividad de la rehabilitación cardiaca (RC) como estrategia de prevención terciaria en la calidad de vida y el control de factores de riesgo de pacientes portadores de cardiopatía coronaria isquémica (CCI) del Instituto Nacional Cardiovascular (INCOR) de EsSalud- Lima, durante el año 2018.

**Materiales y métodos:**

. Se estudió una cohorte retrospectiva de 280 pacientes con diagnóstico de CCI quienes después del tratamiento médico, intervencionista o quirúrgico, fueron derivados al programa de RC de INCOR para prevención terciaria (PT) en el año 2018. El programa se desarrolló según la guía institucional, durante ocho semanas con sesiones de ejercicio y talleres educativos, psicológicos, nutricionales y recreativos. Al inicio y al final de este se les aplicó la prueba de calidad de vida QLMI-2 y se les realizó mediciones antropométricas, laboratoriales y de control de factores de riesgo.

**Resultados.:**

El nivel de calidad de vida al final de la RC mostró una mejoría estadísticamente significativa en las dimensiones emocional, social, física y a nivel global (p < 0,001). Igual comportamiento se observó para las variables nutricionales de peso, circunferencia abdominal e IMC (p < 0,001). La capacidad física mostró una mejora estadísticamente significativa en los aspectos de fuerza muscular (12,2%), actividad física (38,0%) y capacidad funcional (25,4%) (p < 0,001). El resultado no fue homogéneo para las variables bioquímico-metabólicas, donde la hemoglobina glicosilada, la glicemia y el perfil lipídico no mostraron mejoría significativa, con excepción del HDL que elevó sus niveles de manera estadísticamente significativa (p < 0,001).

**Conclusiones:**

. La RC es efectiva como estrategia central para realizar prevención terciaria en los pacientes con CCI ya que mejora ostensiblemente la calidad de vida y los factores de riesgo coronario.

## Abstract (en)

**Objective.:**

Determine the effectiveness of cardiac rehabilitation (CR) as a tertiary prevention strategy in the quality of life and control of risk factors of patients with ischemic coronary heart disease (ICC) of the National Cardiovascular Institute (INCOR) of EsSalud- Lima during the year 2018.

**Materials and methods.:**

A retrospective cohort of 280 patients with a diagnosis of CCI was studied who, after medical, interventional, or surgical treatment, were referred to the INCOR CR program for tertiary prevention (PT) in 2018. The program was developed according to the institutional guide for eight weeks with exercise sessions and educational, psychological, nutritional, and recreational workshops. At the beginning and at the end of this, the QLMI-2 quality of life test was applied, and anthropometric, laboratory, and risk factor control measurements were performed.

**Results.:**

Quality of life levels at the end of CR showed a statistically significant improvement in the emotional, social, physical, and global dimensions (p < 0.001). The same behavior was observed for the nutritional variables of weight, abdominal circumference, and BMI (p < 0.001). Physical capacity showed a statistically significant improvement in the aspects of muscle strength (12.2%), physical activity (38.0%), and functional capability (25.4%) (p < 0.001). The result was not homogeneous for the biochemical metabolic variables, where glycosylated hemoglobin, glycemia, and lipid profile did not show significant improvement, except for HDL, which raised its levels statistically significantly (p < 0.001).

**Conclusions.:**

CR is effective as a central strategy to perform tertiary prevention in patients with ICC since it notably improves quality of life and coronary risk factors.

## Introducción

La cardiopatía coronaria isquémica (CCI) es la primera causa mundial de muerte y carga de enfermedad que, junto al accidente cerebrovascular, representan casi el 80% de la magnitud del problema de salud de las enfermedades cardiovasculares (ECV) ^1^^,^^2^. En el Perú, este grupo de enfermedades ha mostrado un incremento constante en las últimas décadas, pasando de tercera a primera causa de muerte y carga de enfermedad en los mayores de 50 años. Asimismo, la prevalencia de los factores de riesgo cardiovasculares (FRCV) se ha incrementado en los últimos años, configurando un escenario propicio para la propagación y progreso de la CCI en la población peruana ^3^^-^^5^.

El manejo médico actual de los FRCV se basa en el tratamiento farmacológico mas no en la intervención integral de prevención primaria propuesto en guías nacionales e internacionales sobre conducción de estos casos ^6^^,^^7^ lo cual evitaría el progreso de la enfermedad en su historia natural. Consecuentemente, el portador continúa con los procesos fisiopatológicos que generan la obstrucción de las arterias coronarias, ocasionando finalmente la disminución o cese del flujo sanguíneo al miocardio y la consecuente pérdida de la provisión de oxígeno que ocasiona la angina de pecho o el infarto del miocardio ^8^^,^^9^. El panorama descrito, el incremento de la mortalidad y predominancia de la carga de enfermedad de la CCI, se produce pese a la validez de los métodos diagnósticos y la eficacia del manejo integral existente, ergo, la CCI sigue constituyendo un problema de salud pública nacional y mundial ^2^^,^^4^^,^^10^.

En este estado, el paciente con CCI recibe tratamiento para revertir o reducir la obstrucción de las coronarias, intervención que puede ser quirúrgica, intervencionista o farmacológica. La secuencia descrita hasta este punto muestra que los pacientes que han alcanzado este estadio de la CCI han cumplido con dos de los tres estadios reconocidos en la historia natural de la enfermedad; a saber: i) el periodo prepatogénico y, ii) el periodo patogénico. El contexto de desarrollo que describe esta teoría indica que al primer estadio le corresponde la prevención primaria y al segundo la prevención secundaria. Consecuentemente, el tercer estadio (resolución de la enfermedad), debe ser completado con prevención terciaria ^10^^-^^13^, que en el caso del paciente que ha presentado CCI, le corresponde una estrategia reconocida y de probada efectividad como es la rehabilitación cardíaca (RC) ^14^^,^^15^ (Tabla 1).

**Tabla 1:** Historia natural de la CCI, se evidencia las etapas de la enfermedad y su relación con los niveles de prevención y sus estrategias.

Etapa prepatogénica	Etapa patogénica	
Fase exposición	Horizonte clínico- enfermedad	Resolución
Factores de riesgo cardiovascular	Intervenciones médicas	Estrategias recuperativas
Hipertensión arterial	Intervención quirúrgica	Limitaciones de las secuelas
Inactividad física	Tratamiento invasivo hemodinámico	Recuperaciópn del máximo potencial
Estado Nutricional	Tratamiento farmacológico	Reinserción social y productiva
Diabetes		Incremento de la calidad de vida
Síndrome metabólico		
Otros factores reconocidos		
Antecedentes familiares		
Factores epigenéticos		
Factores sociales y ambientales		
Prevención primaria	Prevención secundaria	Prevención terciaria
Manejo integral	Diagnóstico oportuno y prevención de secuelas	Rehabilitación cardíaca
Control farmacológico de los factores de riesgo	Acceso oportuno a la atención	Autoevaluación
Cambio estilo de vida	Prestaciones integrales	Educación nutricional
Hábitos alimentarios	Calidad de la atención	Entrenamiento y formación actividad física
Actividad física		Manejo de la sobrecarga psicológica
Descanso y/o disipación		Adherencia a medidas terapéuticas
		Sostenibilidad y estilo de vida saludable

Elaborado a partir de las referencias 6, 7 y 8

«Como objetivo, la prevención terciaria persigue retrasar el curso de la enfermedad, mejorar las funciones residuales de la persona y minimizar la aparición o gravedad de incapacidad logrando que obtenga el máximo nivel de autonomía, bienestar y calidad de vida» ^16^. Dentro de este marco, la RC se ha erigido como una subespecialidad de la medicina cardiovascular, encargada de la prevención terciaria del paciente cardiópata, cuyo efecto es el control de los FRCV y mejora de la calidad de vida del paciente ^14^^,^^15^. Pese a los logros y posicionamiento reconocidos de la RC, su uso no está suficientemente extendido. En el Perú, el programa más importante lo conduce el Instituto Nacional Cardiovascular «Carlos Alberto Peschiera Carrillo» de EsSalud (INCOR), que aplica de manera integral los modelos de RC reconocidos en la subespecialidad. 

La baja extensión y cobertura de la RC es una situación que debe modificarse, para lo que se requiere generar evidencias sobre su eficacia y efectividad en nuestro medio. Por ello, el objetivo de esta investigación es evaluar cómo la prevención terciaria mediante un programa de rehabilitación cardíaca mejora la calidad de vida y los factores de riesgo de pacientes con ECV, específicamente en los afectados por CCI.

## Materiales y métodos

### Diseño del estudio

La investigación se realizó en una cohorte retrospectiva de pacientes del programa de RC, estableciéndose una medición basal al ingreso y al final del programa. Este se desarrolló en la Unidad Funcional de RC del INCOR según la guía institucional, que establece las condiciones de desarrollo e involucran a un equipo multidisciplinario de profesionales que permiten recuperar las condiciones físicas, mentales, sociales y laborales en pacientes coronarios. Los pacientes reciben evaluación médica cardiológica, física, psicológica y nutricional al inicio y al final del programa; en el interín participan en 24 sesiones de ejercicios tres veces por semana, talleres educativos sobre nutrición, consejería psicológica y actividades recreativas ^17^.

### Población del estudio

La población del estudio estuvo constituida por todos los pacientes mayores de 15 años del INCOR con diagnóstico de CCI, que completaron el programa de RC durante el año 2018. 

### Variables

Las principales variables del estudio fueron calidad de vida, capacidad física, estado nutricional, perfil lipídico y glicemia todas medidas antes y después de completado el programa de RC por los pacientes.

La calidad de vida fue medida mediante el cuestionario Quality of Life After Myocardial Infarction (QLMI 2), en su versión mejorada y validada al idioma español ^18^, consta de veintisiete ítems y tres dimensiones: física, social y emocional. La capacidad física se determinó con dos variables; la primera fue la fuerza muscular medida con un dinamómetro y la segunda la capacidad funcional medido por la ergometría y la prueba de caminata de los 6 min (TC6M). El peso, el IMC (índice de masa corporal) y la circunferencia abdominal, como variables del estado nutricional de los pacientes, se recopilaron a partir de los registros físicos del programa que, según la guía de RC institucional, registró la nutricionista del programa ^17^. Las pruebas de perfil lipídico y glicemia fueron realizadas por el servicio de laboratorio clínico contratado por el INCOR para el servicio de apoyo al diagnóstico.

Las variables complementarias como la presencia de FRCV (hipertensión arterial [HTA], diabetes *mellitus*, dislipidemia, consumo de tabaco, obesidad, antecedente de ECV) y tratamiento farmacológico del paciente, fueron recopilados de los registros clínicos institucionales, principalmente la historia clínica electrónica y sus complementos físicos, por la investigadora principal y la psicóloga del equipo de RC, quienes realizaron la digitación en una hoja de cálculo del programa Excel®.

### Procesamiento y análisis de datos

La calidad de los datos fue evaluada mediante análisis descriptivo de la correspondencia y consistencia de los datos. Las inconsistencias observadas fueron contrastadas con los registros correspondiente, procediéndose a su corrección.

Para el análisis descriptivo se obtuvieron las distribuciones de frecuencias absolutas y relativas (variables categóricas) y medidas de tendencia central y dispersión de acuerdo con el comportamiento normal, o no, de la variable numérica. En la segunda fase se determinó el grado de normalidad de las distribuciones de las variables mediante la prueba de Shapiro-Wilk, proceso realizado con el programa SPSS v 22 ^19^.

Para determinar la significación estadística de los cambios de la calidad de vida previa y posterior a la RC y de las variables estudiadas, se utilizó la Prueba de los rangos con signo de Wilcoxon; debido a que la distribución de todas las variables no resultó en una distribución normal (p < 0,001 en todos los casos). En este análisis se utilizó el programa JASP v 0.18.1 ^20^.

### Aspectos éticos

El estudio fue aprobado el 9 de marzo del 2020 mediante la Nota N.º 32-CE-DIR-INCOR-ESSALUD-2020, por el Comité Institucional de Ética en Investigación del INCOR.

## Resultados

Durante el 2018, los 280 pacientes con CCI que ingresaron al programa de RC del INCOR fueron incorporados al estudio; la edad mínima fue de 31 años y la máxima de 85 años. El 88,6% correspondieron al sexo masculino, cuya media de edad fue 64,4 años, mientras que para las mujeres fue de 62,3 años.

En cuanto a la distribución de los FRCV se observó en todos los casos proporciones elevadas. La HTA fue el factor de mayor predominancia seguido por el antecedente de ECV, la dislipidemia y el antecedente de tabaquismo. La distribución según sexo no mostró mayores diferencias, excepto para la dislipidemia que mostró mayor prevalencia para las mujeres y el consumo de tabaco que fue mayor en los varones (Tabla 2).

**Tabla 2:** Distribución de los factores de riesgo según sexo. Programa de Rehabilitación Cardiaca. INCOR, 2018

Antecedentes y factores de riesgo	Masculino (n = 248)		Femenino (n = 32)		Total	
	n	%	n	%	n	%
HTA	154	62,1	21	65,6	175	62,5
DM2	72	29,0	8	25,0	80	28,6
Dislipidemia	110	44,4	19	59,4	129	46,1
Consumo de tabaco	108	43,5	12	37,5	120	42,9
Obesidad	49	19,8	7	21,9	56	20,0
Antecedente de ECV	119	48,0	16	50,0	135	48,2

HTA: hipertensión arterial. DM2: diabetes mellitus tipo 2. ECV: enfermedad cerebrovascular

Con relación al tratamiento para la CCI, el 66,8% tuvo una angioplastia coronaria, un 21,8% fue sometido a revascularización quirúrgica, el 7,9% tuvo solo manejo farmacológico y el 3,6% recibió revascularización híbrida. 

Al iniciar el programa, y como parte del tratamiento farmacológico de la CCI, los pacientes recibieron una media de 4,9 medicamentos/paciente, destacando que el 61,8% recibieron cinco o más medicamentos. Los medicamentos más frecuentemente prescritos fueron los betabloqueadores (90,7%), los antiagregantes plaquetarios (95,4%) y doble agregación antiplaquetaria con 74,3% (Tabla 3).

**Tabla 3:** Distribución de medicamentos administrados a los pacientes del Programa de Rehabilitación Cardiaca. INCOR, 2018

Medicamentos prescritos	n	%
Ácido acetilsalicílico	267	95,4
Atorvastatina	266	95,0
Betabloqueadores	254	90,7
Clopidogrel	208	74,3
Bloqueadores angiotensina II	114	40,7
Warfarina	63	22,5
Inhibidores de la ECA	59	21,1
Nitratos	41	14,6
Metformina	41	14,6
Espironolactona	28	10,0

ECA= enzima convertidora de angiotensina

### Factores de riesgo cardiovascular

Los valores del perfil lipídico y de la glucosa, si bien mostraron disminución al final de la RC, esta no fue estadísticamente significativa, con excepción de los niveles de HDL (p < 0,001) (Tabla 4).

**Tabla 4:** Distribución comparativa de las variables del perfil bioquímico, nutricionales y de capacidad física de los pacientes del Programa de Rehabilitación Cardiaca. INCOR, 2018

Variables	Mediana		Valor p ^*^
	Pre-RC	Pos-RC	
Perfil bioquímico			
Hemoglobina glicosilada (mg/dL)	6,0	5,8	1,000
Glucosa (mg/dL)	103,0	100,0	1,000
Colesterol total (mg/dL)	118,0	117,5	0,073
HDL (mg/dL)	37,0	40,0	< 0,001
LDL (mg/dL)	52,8	53,0	0,071
VLDL (mg/dL)	21,5	20,8	0,996
Triglicéridos (mg/dL)	108,0	106,5	0,986
Nutricionales			
Peso (kg)	73,0	71,6	< 0,001
Índice de masa corporal	26,87	26,20	< 0,001
Circunferencia abdominal (cm)	96,0	93,9	< 0,001
Capacidad física			
Fuerza muscular (kg)	29,0	32,0	< 0,001
Distancia recorrida (metros)	334,0	459,0	< 0,001
Capacidad funcional (METS)	5,97	7,43	< 0,001

RC= rehabiilitación cardíaca

* Prueba de los rangos con signo de Wilcoxon

Con relación a las variables nutricionales evaluadas, el peso, la circunferencia abdominal y el IMC redujeron sus valores de forma estadísticamente significativa, aunque con disminuciones menores al 3,0% entre la medianas previa y posterior a la RC (Tabla 4).

Para las variables de la capacidad física, la RC también produjo cambios favorables y estadísticamente significativa (p < 0,001). La fuerza muscular mejoró en un 10,3% comparando el valor basal con el final. El tiempo de actividad física mejoró en un 37,4% en el mismo sentido, y la capacidad funcional mejoró en un 24,4%; todos calculados a partir de las medianas previas y posteriores a la RC (Tabla 4).

### Calidad de vida

La media de las dimensiones de la calidad de vida en el pos-RC se incrementaron en 10,3; 13,6 y 9,5 en el aspecto emocional, físico y social, respectivamente, con relación a la medición basal. Para el indicador global del QLMI 2, el incremento alcanzó los 33,5 puntos.

Para determinar si el cambio descrito obtenido se relacionaba con la participación de los pacientes en la RC, se aplicó la prueba de los signos con rango de Wilcoxon (p < 0,001), pues, como se indicó en materiales y métodos, la distribución no se ajustó a la distribución normal de acuerdo con la prueba de Shapiro (p < 0,001).

**Figura 1:**
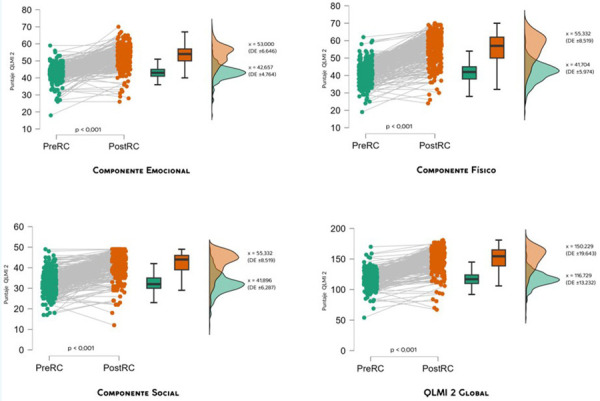
Efectividad de la prevención terciaria en las dimensiones de la calidad de vida medidas con el QLMI-2 en los pacientes del programa de Rehabilitación Cardiaca. INCOR, 2018.

En este sentido, la prueba de Wilcoxon mostró resultados estadísticamente significativos para las dimensiones emocional, física y social, así como para el indicador global de la calidad de vida (p < 0,001) (Figura 1).

La significación obtenida en la medición de la calidad de vida con el QLMI 2 muestra claramente que el programa de RC es sustancialmente efectivo para influir en la historia natural de la CCI. Sin embargo, como puede observase en el *Raincloud plot* de la Figura 1 (primera representación de la izquierda de cada componente), algunos casos no mostraron mejoría e incluso algunos mostraron una disminución posterior a la RC. Este resultado estaría relacionado con la necesidad de extender la duración de la RC, según la evaluación que se efectúe a cada paciente durante el programa.

## Discusión

Este estudio en 280 pacientes con enfermedad coronaria encontró que la RC, como estrategia de prevención terciaria, mejora los parametros de calidad de vida medidos con el cuestionario QLMI2 y de los factores de riesgo coronario al finalizar el programa de sesiones de RC.

El resultado descrito se sustenta en la mejora de los componentes: emocional, físico y social que resultan en incremento de la calidad de vida de los pacientes estudiados, independientemente del tratamiento recibido. Consecuentemente, la RC debe ser indicada a todo paciente portador de este tipo de enfermedad, tal como se muestra en estudios similares ^21^^,^^22^.

El cambio observado, aunque se dio en los tres componentes, fue comparativamente menor en el aspecto social relacionado con el tiempo que requiere el paciente y su familia para retomar a cabalidad su vida social, así como la corta duración del programa de RC en el INCOR, insuficiente para lograr cambios de hábitos personales y familiares que sostengan el cambio de estilo de vida que busca la RC ^16^^,^^23^^-^^25^.

Nuestros resultados corroboran los obtenidos en diversos estudios, mostrando que la RC, independientemente de las diferentes formas de abordaje, de aplicación o duración que tenga, termina evidenciando los beneficios para el paciente. Para el caso específico de nuestra investigación, el enfoque retrospectivo aplicado, cuyas limitaciones se corresponden al de todo estudio que utiliza fuentes secundarias; no diluye o altera el efecto benéfico que la RC brinda al paciente cardiópata, agregando mayor consistencia al conocimiento existente sobre la efectividad de esta estrategia de la prevención terciaria ^14^^,^^23^^-^^27^.

La fortaleza de un programa de RC para lograr la efectividad descrita en diversos estudios y en esta investigación, está asociada a su abordaje integral y específico que realiza. Así, tenemos que el buen control nutricional, obtenido a partir de la prescripción de ejercicios y orientación nutricional, resulta en una mejora del peso y el IMC del paciente, mejorando el control de la obesidad. Nuestro programa de RC ha logrado este cambio que es compatible con lo descrito sobre este componente en otros programas ^28^^-^^30^.

Nuestro estudio muestra que la capacidad física mejora notoriamente con la RC, destacando dentro de los tres componentes, el de la capacidad funcional. En este caso, el incremento de METS del 25,4% obtenida al final con relación al basal, muestra claramente la mejora de la capacidad física. La literatura científica también describe que este componente está interrelacionado, pues la capacidad funcional está asociada a una mayor duración de la actividad física y la fuerza muscular de los pacientes cardiópatas, variables cuya mejora se evidenció en nuestros resultados ^31^^-^^33^.

Los aspectos metabólicos estudiados también mostraron mejora, aunque no mostraron significación estadística. Tanto los niveles de glucosa, hemoglobina glicosilada, como los lípidos en sangre, mostraron variaciones hacia sus valores normales, variaciones de poca magnitud relacionadas con la duración de dos meses y una media de 21,1 sesiones por paciente, cantidad insuficiente, considerando que la mayoría de los programas de RC se prolongan por encima de los 3 meses y hasta doce meses, dependiendo de la disponibilidad del servicio y de la evaluación que se haga del paciente ^33^^-^^36^. Esta limitación se relaciona con la poca oferta de servicios de RC, que en el caso del INCOR, al momento de la publicación del estudio, este era el único programa nacional con todos los componentes y logística correspondiente, lo que limita la cobertura de la prevención terciaria para la CCI y las ECV ^28^^,^^37^^,^^38^. Esta situación no se relaciona solo con la poca oferta de estos servicios, sino también con enfoques y perspectivas inadecuadas de los médicos cardiólogos y no cardiólogos ^39^^-^^41^.

La premisa y contexto descrito resultan de gran importancia, si consideramos que proporciones variables de médicos no refieren a sus pacientes a los programas de RC pese al probado beneficio que brinda a los pacientes cardiópatas. En este escenario, la difusión entre los médicos cardiólogos y no cardiólogos, del enfoque de prevención terciaria como parte del proceso de la historia natural de la CCI y de la continuidad de sus estadios, así como su relación con los niveles de prevención primaria y secundaria, resultará de gran utilidad , no solo para mejorar la referencia y utilización de los servicios de RC, sino además para impulsar la creación y puesta en funcionamiento de más servicios de RC, que como se ha indicado, es la estrategia por definición de la prevención terciaria para la CCI ^7^^,^^11^^,^^37^^,^^42^.

Dentro de las limitaciones del estudio, la principal es la que corresponde a todo estudio de fuentes secundarias que se caracterizan por la heterogeneidad del registro y la necesidad de utilizar fuentes complementarias para integrar la data faltante. En este estudio la recopilación de datos pudo completarse por la facilidad de los investigadores para acceder a registros complementarios del programa.

En conclusión, la RC es efectiva como estrategia central para realizar prevención terciaria en los pacientes con CCI ya que mejora ostensiblemente la calidad de vida y los factores de riesgo coronario y la propuesta final de este estudio, que esperamos sea integrada a los procesos de formación de especialistas y a los programas de educación médica continua, es que la prevención terciaria es la etapa siguiente, obligatoria y beneficiosa de todo paciente que ha recibido prevención secundaria (diagnóstico oportuno y tratamiento temprano) por CCI, incorporación basada en los consistentes resultados de la efectividad de la RC para mejorar la evolución y el pronóstico de un paciente con CCI.
